# Varicosity of Vortex Vein Ampulla in Ocular Fundus: Descriptive Series of 53 Patients and Literature Review

**DOI:** 10.3390/jcm15103614

**Published:** 2026-05-08

**Authors:** Jonathan T. Regenold, Zélia M. Corrêa, Robert H. Osher, James J. Augsburger

**Affiliations:** 1 Department of Ophthalmology, University of Cincinnati Academic Health Center, 231 Albert Sabin Way, Cincinnati, OH 45267, USA; jonathanregenold@gmail.com (J.T.R.); augsbujj@ucmail.uc.edu (J.J.A.); 2 Bascom Palmer Eye Institute, University of Miami Miller School of Medicine, 900 NW 17th St. Suite 261, Miami, FL 33136, USA; 3 Sylvester Comprehensive Cancer Center, University of Miami Miller School of Medicine, 900 NW 17th St. Suite 261, Miami, FL 33136, USA; 4 Cincinnati Eye Institute, 1945 CEI Drive, Cincinnati, OH 45242, USA; rhosher@cvphealth.com

**Keywords:** ampulla, varicosity, varix, vortex vein, choroidal mass

## Abstract

**Background/Objectives**: Varicosities of the vortex vein ampulla are transient dilations of vortex vein ampullae that appear as red-brown choroidal masses. The purpose of this manuscript is to describe a retrospective case series of 53 patients with varicosities of the vortex vein ampulla and perform a literature review on this topic. **Methods**: Our case series demonstrates the clinical features of a large cohort of varicosities of the vortex vein ampulla, including their locations in the ocular fundus, sizes when congested, direction of gaze that resulted in detection, frequency of multiple lesions in a single eye, and frequency of bilateral cases. The literature review utilized PubMed and Embase libraries and included all studies published through December 2025. **Results**: The literature review yielded 44 articles, of which 36 were deemed relevant. Several studies described the appearance of these lesions using imaging modalities, including B-scan ultrasonography, optical coherence tomography, and indocyanine green angiography. Others underscored the potential for these lesions to be mistaken for other types of choroidal masses, such as choroidal melanomas. **Conclusions**: This extensive series demonstrates that these lesions are most often located nasally, sometimes multiple or bilateral, and often mistaken for choroidal nevi or melanomas, highlighting the importance of understanding clinical characteristics for appropriate diagnosis. In addition, some studies described possible associations with conditions such as nodular scleritis and Donnai–Barrow syndrome.

## 1. Introduction

Varix or varicosity of a vortex vein ampulla in the ocular fundus was first described as a distinct clinical entity by Osher et al. in 1981 [1]. The affected vortex vein ampulla becomes visibly congested and darker when the patient looks in a particular direction of gaze during ophthalmic examination. Its elevation when congested can be confirmed in many cases by diagnostic ocular ultrasonography or optical coherence tomography (OCT), as well as sometimes by fundus photography; however, because of its typical position near the ocular equator [2,3], good photographic documentation is frequently difficult. Although varicosity of a vortex vein ampulla is not known to cause any functional abnormalities of the affected eye, it is occasionally mistaken for a choroidal nevus or small choroidal melanoma by inexperienced examiners performing indirect ophthalmoscopy [4].

Recent studies have examined vortex vein ampulla varicosities using multimodal imaging [5,6]. Veronese et al. [5] performed a retrospective analysis of eight patients, highlighting the utility of multimodal imaging in characterizing these lesions. Reported findings include hyporeflective lesions on infrared imaging, hyporeflective cavities with internal lobules using extended depth imaging optical coherence tomography, and hyperfluorescence with progressive pooling using indocyanine green angiography (ICGA). While multimodal imaging may aid in differentiating vortex vein ampulla varicosities from other choroidal lesions, most lesions of this type do not require ancillary testing for diagnosis. The purpose of our study was to perform a literature review of papers on this topic and to describe clinical features of vortex vein ampulla varicosities in a large cohort that may provide additional differentiating features without reliance on advanced imaging.

## 2. Materials and Methods

The authors performed a retrospective descriptive subgroup study of patients found to have one or more fundus lesions consistent with varicosity of the vortex vein ampulla who were encountered in a single ocular oncology practice during a 40-year interval (1979–2018). The principal aspects of these lesions evaluated in this study were (1) the proportion of such lesions that had been detected by an eye care professional and prompted referral to our ocular oncology practice versus detected during ophthalmoscopy in our practice performed for an unrelated abnormality, (2) the frequency of multiple such lesions in an affected eye and patient, (3) the spectrum of size of such lesions when congested, (4) the frequency distribution of topographical location of the lesions in the ocular fundus, (5) the prevalence of bilateral cases, and (6) the gaze direction of the eye that prompted congestion of the identified vortex vein ampullae.

The 53 cases evaluated in this study were those of patients diagnosed as having one or more fundus lesions consistent with varicosity of the vortex vein ampulla encountered in the clinical ocular practice of authors JJA and ZMC. Lesions were considered vortex vein ampulla varicosities if they demonstrated gaze-induced congestion, a feature considered pathognomonic for this lesion, and the absence of clinical features suggestive of choroidal nevus or melanoma, such as associated subretinal fluid or drusen [7]. While collapse with digital pressure is a known characteristic, it was not consistently documented in the charts included in this series so was not used as a mandatory inclusion criterion. One outlier case that demonstrated persistent congestion despite change in gaze direction was included as it demonstrated features highly suggestive of vortex vein ampulla varicosity, including confluence with the vortex vein system and lack of features suggestive of choroidal hemangioma and other mimicking lesions. All known patients with vortex vein ampulla varicosities evaluated by either clinician prior to initiation of the study were included. These cases were identified by review of a comprehensive diagnostic database of all patients who had been encountered in the practice that had been prepared by the senior author during 2023. No cases were included between 2018 and 2023, as the senior author (JJA) retired from clinical practice in 2018. Prior to data abstraction, a list of variables was created to facilitate data abstraction form that included all the relevant raw variables. Included on this list was the diameter of such lesions when congested.

The dimensions (largest basal diameter, smallest basal diameter, and thickness) of all lesions evaluated in this study were estimated by a single examiner (JJA). Chord length basal diameters of the evaluated lesions were estimated to the nearest 0.5 mm during binocular indirect ophthalmoscopy performed using condensing lenses of different dioptric powers and diameters. The diameter of the visualized portion of the ocular fundus when the virtual image filled the lens was used as the scale for these estimates. The proportion of the diameter of the visualized field occupied by the lesion of interest determined these estimates. Arc length basal diameters of the evaluated lesions were also estimated for each lesion, also to the nearest 0.5 mm, by performing cartographical analysis of fundus drawings prepared using azimuthal equidistant projection map charting forms. Available fundus photos of the lesions and contemporaneous annotations made by the examiner at the time the fundus drawing was prepared were used to confirm the accuracy of the fundus maps. The arc lengths of largest and smallest basal diameter of each lesion were converted to chord lengths using an arc length to chord length conversion table that took into account the approximate outside diameter of the eyeball. The chord lengths of individual basal lesion dimensions estimated directly by indirect ophthalmoscopy and those estimated indirectly by arc length to chord length conversion from the fundus drawings were reconciled as the average of the two estimates, expressed to the nearest 0.5 mm. These estimations were corroborated by B-scan ultrasound when available In most cases, thickness measurements of the lesion when congested were corroborated by ocular ultrasonography.

The first author (JTR) reviewed each ocular oncology chart of the identified patients and collated the preplanned information from the charts onto the data abstraction forms. The first author then transcribed the information from the data abstraction forms into an electronic data analysis database (SPSS^®^ for Windows, Version 8.0.0, IBM Corp., Armonk, NY, USA). The authors performed descriptive data analysis of the evaluated variables and tabulated the summary information on these cases.

### Literature Review

A literature search was performed using PubMed and Embase. All articles published online through December 2025 were included. The search terms used were “vortex vein ampulla varicosity”, “vortex vein ampulla varix”, “vortex vein varicosity”, and “vortex vein varix”. No language restrictions were applied; however, for articles not published in English, data abstraction was limited to manuscripts translated to English via translation software (Google Translate; Google inc., Mountain View, CA, USA) and already available English-translated abstracts. In these cases, certain clinical variables were not always available for assessment.

Inclusion criteria consisted of relevant clinical articles describing vortex vein ampulla varicosities, while exclusion criteria included any article that did not describe clinical cases of the lesion of focus, such as orbital varices or vortex vein anomalies that were not varicosities. Collected articles were assessed for clinical variables of patients including the number of vortex vein ampulla varicosity lesions, eyes involved, lesion location, multiplicity and bilaterality of lesions, dynamic behavior of lesions, multimodal imaging of lesions, and conditions possibly associated with lesions. For studies involving multiple patients or lesions, the lesion location, multiplicity, and bilaterality of lesions were summarized with descriptors rather than individually.

## 3. Results

### 3.1. Case Series

Our retrospective study group consisted of 53 patients. A summary of evaluated clinical variables and their categories and scale units are presented in Table 1. In total, 34 patients (64.2%) were men and 19 (35.8%) were women. The youngest patient was 19.6 years old at initial diagnosis and the oldest was 85.6 years old. The median patient age was 58.9 years. A total of 14 of the 53 patients (26.4%) were referred to our practice because of a detected varicosity of the vortex vein ampulla. The referral diagnosis was choroidal nevus, choroidal melanoma, or “rule out” choroidal melanoma in all these cases. In contrast, 39 patients (73.6%) were found to have one or more varicosities of the vortex vein ampulla following ophthalmoscopy performed in our ocular oncology practice, even though these lesions had not been noted by the referring doctor. None of these patients had any ocular symptoms attributable to a vortex vein ampulla lesion or any visual impairment related to such lesion.

In total, 46 of the 53 patients (86.8%) had a single varicosity of the vortex vein ampulla in one affected eye, four patients (7.5%) had two varicosities of the vortex vein ampulla in one affected eye, two patients (3.8%) had one varicosity of the vortex vein ampulla in each eye, and one had two varicosities of the vortex vein ampulla in each eye. The right eye only was affected in 29 patients (54.7%), the left eye only was affected in 21 patients (39.6%), and both eyes were affected in three patients (5.7%). The total number of clinically identified varicosities of the vortex vein ampulla in the 56 affected eyes of the 53 study patients was 62.

**Figure 1:**
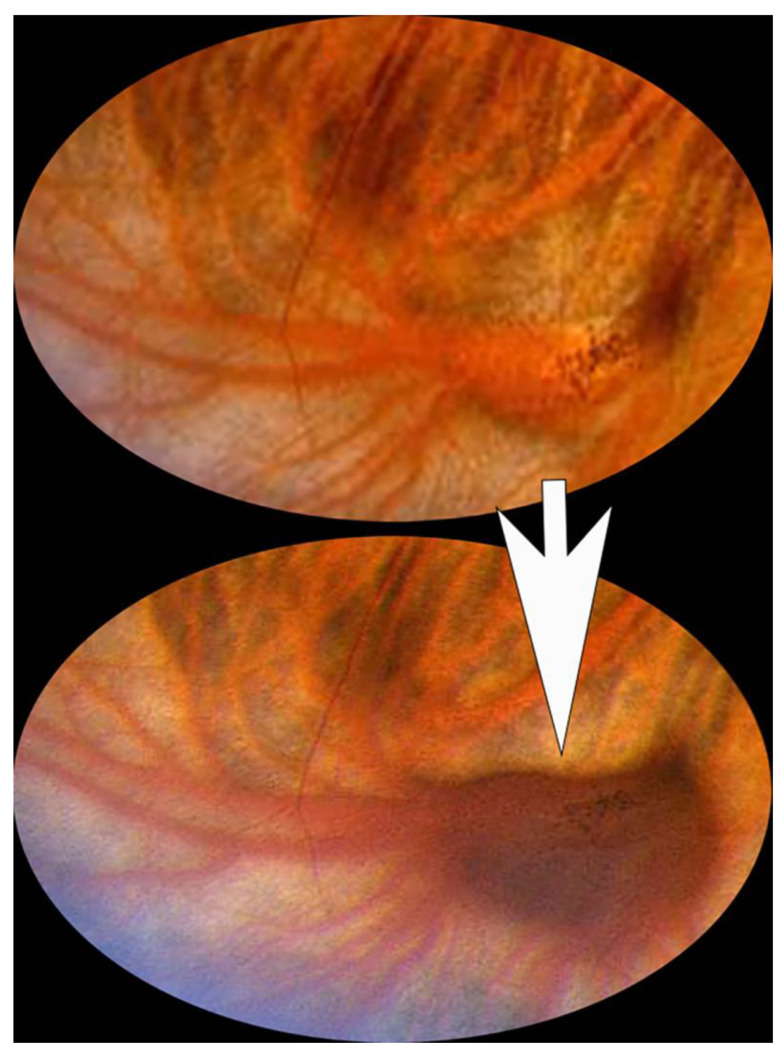
Magnified fundus photo showing a vortex vein ampulla varix in a detumescent state (**top**) and a congested state (**bottom**). This transition was observed clinically as the patient’s direction of gaze was changed toward the affected quadrant.

**Figure 2:**
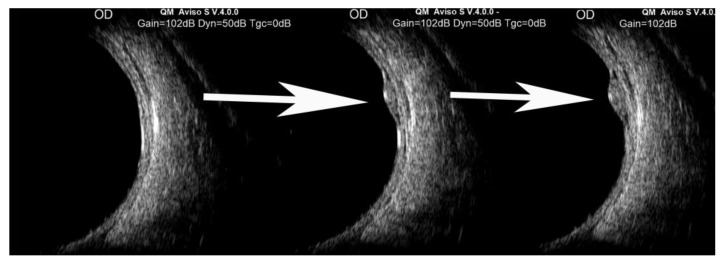
A series of three sequential B-scan ultrasound images capturing intumescence of a vortex vein ampulla varix. During clinical exam, this was observed dynamically as light digital pressure was released from the pre-equatorial quadrant where the lesion was located.

The distribution of the varicosities is summarized in Table 1. In total, 24 of the 62 (38.7%) clinically identified varicosities of the vortex vein ampulla were located in the superonasal quadrant of the ocular fundus and another 24 (38.7%) were in the inferonasal quadrant (Figure 1), while only 10 (16.1%) were inferotemporal and only four (6.5%) were superotemporal. The median measurements of the vortex vein ampullae when congested were as follows: largest basal diameter = 3 mm (minimum 1.5 mm, maximum 8.0 mm); smallest basal diameter = 2 mm (minimum 0.5 mm, maximum 4.5 mm); and median height = 1 mm (minimum 0.5 mm, maximum 1.5 mm) (Figure 2).

The relationship between the direction of gaze that resulted in congestion of vortex vein ampulla and the topographical fundus lesion location of the affected vortex vein ampulla is shown in Table 2. In most cases (49 of 61, 80.3%), the direction of gaze that resulted in congestion of the vortex vein ampulla was toward the quadrant of the fundus lesion. Interestingly, one patient exhibited a lesion in the superonasal quadrant of the right eye that appeared slightly congested persistently regardless of the direction of gaze. This lesion was not associated with other ocular pathology but was among the largest in the series, measuring 7.0 mm in its largest basal diameter, and exhibited a markedly irregular basal shape. No lesion in this series was evaluated by FA, ICGA, OCT-A, fundus autofluorescence, or other multimodal imaging methods.

### 3.2. Findings of Literature Review

Our literature search yielded 44 publications, of which 36 were deemed relevant based on the inclusion/exclusion criteria (Table 3). The eight excluded articles were deemed irrelevant as they described pathologies other than the lesion of focus (e.g., orbital varices or vortex vein anomalies that were not true varicosities). Five articles were not originally published in English (Table 3). Data from these articles was abstracted from English-translated abstracts or original manuscripts translated via language translation software when available.

Across these studies, most lesions were unilateral and singular, although bilateral and multiple lesions were infrequently reported. The most common reported dynamic behavior was collapse with globe pressure (25 studies [69.4%]) while the second most common was gaze-evoked intumescence (15 studies [41.7%]). The least common reported dynamic behavior was valsalva-induced intumescence (6 studies [16.7%)].

Gaze-evoked intumescence and collapse with globe pressure were the most commonly reported dynamic behaviors. These lesions were occasionally identified as mimickers of more serious pathologies, including choroidal melanoma and subretinal hemorrhage. They were also reported in association with systemic and ocular conditions, including Donnai–Barrow syndrome and nodular scleritis. A majority of recent studies focus on advanced imaging modalities, including OCT and ICGA, to further characterize these lesions.

## 4. Discussion

In their initial description of lesions of the type we evaluated in our study, Osher et al. [1] identified most of the relevant currently recognized features of such lesions, including the following: (1) abrupt congestion and just as abrupt collapse (detumescence) of the affected vortex vein ampulla depending on the patient’s direction of gaze; (2) congestion of the affected vortex vein ampulla induced by direction of gaze towards the quadrant where the vortex vein ampulla is located; (3) collapse of the congested vortex vein ampulla when light digital pressure is applied to the eye during ophthalmoscopy; (4) congestion of the vortex vein ampulla induced by patient inversion or Valsalva maneuver even when the primary position of gaze is maintained; (5) the change in internal to external dimension (height) of a vortex vein ampulla when the ampulla is congested versus collapsed were documented by ultrasound (Figure 1 and Figure 2); (6) normal filling and draining of the affected vortex vein ampulla when evaluated by fluorescein angiography (FA); and (7) potential for bilateral involvement.

Additional features described by subsequent authors on this topic include variable size of the affected vortex vein ampulla when congested versus collapsed documentable by optical coherence tomography (OCT) [6] and multiplicity of vortex vein ampullae that become congested in some eyes [7,8].

Our series of cases of this entity (53 patients, 56 affected eyes, 62 lesions) is the largest series yet reported. The largest previously reported series consisted of 22 patients (22 affected eyes, 22 lesions) [9]. This series emphasizes the utility of multimodal imaging, such as real-time OCT, in identifying the dynamic vascular nature of these lesions. While their study corroborates our finding that these lesions predominately appear nasally, our series contributes additional information not previously reported in the literature, including the direction of gaze that induces lesion intumescence, and multiplicity, bilaterality, and approximate dimensions of these lesions. Furthermore, every patient in the Levin et al. [9] series exhibited a unifocal, unilateral presentation, whereas we observed bilateral involvement in 5.7% of patients and multiple lesions in a single eye in 9.4% of patients.

In our series, the affected vortex vein ampulla was most often located in the oblique nasal quadrants of the fundus (i.e., superonasally or inferonasally) and infrequently in the oblique temporal quadrants (i.e., superotemporally or inferotemporally). None of the lesions was in the direct vertical or horizontal meridians of the fundus. All the lesions in our series were located in the posterior portion of the equatorial zone of the peripheral fundus [39]. Although one might speculate that some of these lesions might occur secondary to compression of the vortex vein at or near its exit from the sclera by an extraocular muscle or its tendon when the eye looks in a particular direction, such a mechanism seems unlikely in most cases. Kinking of the vein in the orbit at or near its scleral exit site seems to be a more satisfactory explanation for most lesions of this type [10]. To our knowledge, there is no satisfactory anatomic explanation for the preferential location of these lesions in the oblique nasal quadrants.

Varix of a vortex vein ampulla is generally considered to be quite rare [11,12,13]; however, to our knowledge, no report of the frequency of this lesion based on a prospective study of normal eyes of healthy persons with differing levels of refractive error and different axial lengths (let alone of eyes or patients affected by specified disorders) has ever been published. Even in eyes that exhibit such a direction-of-gaze induced transient congestion of a vortex vein ampulla, most vortex vein ampullae in that eye are not affected. Reports of a few eyes that exhibited two separate varicosities of vortex vein ampullae in a single eye have been published [5,7,8] as well as an occasional report of one or more lesions of this type in both eyes [8]. In our series, two patients (3.8%) had one varicosity of the vortex vein ampulla in each eye and one (1.9%) had two varicosities of the vortex vein ampulla in each eye.

One of the most clinically relevant findings of our study is that 39 of 53 patients (73.6%) were diagnosed with a vortex vein ampulla varix incidentally during examination for an unrelated condition. This substantial rate suggests that these lesions go routinely unnoticed in clinical practice and, while rarely reported, are likely much more prevalent in the general population. These lesions often remain undetected because they typically engorge only in a specific direction of gaze and may therefore appear as a normal, collapsed vortex vein ampulla during fundoscopic examination. Furthermore, clinicians may naturally bias their examination towards looking for posterior pole pathologies and may not perform as detailed of an examination of the mid- and peripheral retina. Clinicians may incorporate dynamic gaze changes during indirect ophthalmoscopy to increase the likelihood of detecting these lesions. In our retrospective study, few charts contained information about the impact of light digital pressure on the globe or Valsalva maneuver on the gaze-induced overfilling of the affected vortex vein ampullae. Additionally, charts did not contain information on the refractive error of the affected eyes or the ocular axial length of those eyes. Because of this, none of these variables were evaluated in this study. None of the patients in this series had been subjected to dynamic orbital vascular imaging by computed tomographic or magnetic resonance imaging methods to evaluate the precise cause of the gaze-induced congestion of the affected vortex vein ampullae.

A varix or varicosity of a vein is generally defined as a persistently dilated venous blood vessel due to chronically elevated intraluminal venous pressure. Classic examples are varicose veins of the lower extremities and esophageal varices. While bodily inversion, where the legs are elevated above the heart, can result in a reduction in intraluminal venous pressure within varicose veins of the lower extremities, the affected veins remain dilated, albeit less congested. The transient congestion of a vortex vein ampulla induced by gaze direction described in this report does not, in our opinion, conform to the definition of a true varix. The affected vestibule or ampulla of a choroidal vortex vein becomes overfilled due to transient increased intraluminal venous pressure in that system, which is presumably attributable to partial mechanical obstruction of a vein connecting the ampulla to either the superior or inferior ophthalmic vein in the orbit after its exit from the sclera. As soon as the eye looks in an alternate direction of gaze, the intraluminal venous pressure falls, and the overfilling of the ampulla disappears. In our opinion, there is currently no existing term that adequately describes this lesion.

One vortex vein ampulla in our series was slightly congested regardless of the eye’s direction of gaze. This vortex vein ampulla exhibited a large, markedly irregular basal shape different from that of any other ampulla in the series. This feature suggested to us that this vascular lesion may have resulted from persistent partial obstruction of the vortex vein connecting it to the superior ophthalmic vein in the orbit resulting from anomalous venous variation. Because this ampulla exhibited persistent dilation, it might be classified appropriately as a true varix of that ampulla. Buettner et al. [4] similarly described a patient with an engorged vortex vein ampulla varix that persisted despite changes to the patient’s direction of gaze or body positioning. This lesion did disappear with sufficient globe pressure. The authors hypothesized that this persistent engorgement was due to a very narrow emissary scleral canal, preventing venous outflow from the varix. In summary, this study can be criticized by its retrospective design, but it appears to be the largest reported series of vortex vein ampulla varicosities demonstrating their approximate sizes, direction of gaze resulting in engorgement, occasional multiplicity or bilaterality, and most often nasal distribution. These lesions are clinically significant as they may mimic choroidal melanoma or nevus. Awareness of the described clinical features and dynamic behavior of these lesions may help clinicians reach an accurate diagnosis.

### 4.1. Literature on Clinical Features of Vortex Vein Ampulla Varices

Review of existing literature on the topic of vortex vein ampulla varices demonstrated that collapse with globe pressure was the most reported dynamic finding (69.4% of included studies), followed by gaze-evoked (44.4% of included studies) and Valsalva-evoked intumescence (16.6% of included studies). This discrepancy underscores the heterogeneity in techniques used to diagnose these lesions. We suspect the high reporting of collapse with globe pressure is secondary to the common use of B-scan ultrasonography to aid in diagnosis, which incidentally demonstrates lesion collapse during probe use. Conversely, Valsalva is rarely utilized in clinical environments. The reporting frequency of gaze-evoked intumescence being lower than that of collapse with globe pressure likely reflects a reporting bias, where authors may have not deemed it necessary to explicitly report as gaze-evoked intumescence is considered pathognomonic for this entity.

Of the 36 included studies, the most frequent mimicking conditions were choroidal melanoma (n = 8, 21.6%) and choroidal or subretinal hemorrhage (n = 3, 8.1%). Other mimickers included choroidal metastases (n = 2, 5.4%) and choroidal nevus (n = 1, 2.7%). The observation that a substantial number of cases were originally mistaken for serious pathologies such as ocular cancer highlights the importance of being able to differentiate these lesions. Fortunately, with the application of clinical maneuvers, such as dynamic gaze changes and Valsalva, and multimodal imaging, these lesions can be reliably differentiated from vortex vein ampulla varices.

### 4.2. Literature on the Topic of Multimodal Imaging

Recent studies have utilized multimodal imaging to study these lesions. On color fundus photography, vortex vein ampulla varicosities most often appear as maroon-colored choroidal masses, though nonpigmented lesions have also been described [14,40]. Near-infrared reflectance imaging shows a hyporeflective lesion that is clearly delineated from surrounding structures [9].

Indocyanine green angiography (ICGA) provides valuable information about the choroidal circulation and clearly highlights vortex vein ampullae [11]. Vortex vein ampulla varicosities demonstrate early, homogenous pooling of dye without associated leakage or staining. Applied ocular pressure or changes in gaze, to where the lesion is no longer intumescent, results in a gradual reduction in hypercyanescence. Shields et al. reported dye filling of a vortex vein ampulla varicosity beginning at 38 s following administration of indocyanine green and reaching maximum filling at 70 s [15]. Despite the ability of ICGA to clearly delineate these lesions, this imaging modality is limited in its clinical utility given the invasive nature of the procedure and ability to discern these lesions based on clinical exam.

Fluorescein angiography, by contrast, provides little additional information, given that vortex vein ampulla varices may only appear mildly hyperfluorescent within a background of choroidal flush [5].

B-scan ultrasonography demonstrates a dome-shaped elevation with low internal reflectivity. The lesion flattens when pressure is applied to the eye or the patient’s gaze is redirected, so that the lesion is no longer intumescent [16].

Optical coherence tomography (OCT) shows elevation of the retinal pigment epithelium corresponding to the lesion with underlying hyporeflectivity [14,17]. It also demonstrates intact lamination of the retinal layers with no associated subretinal or intraretinal fluid. Adjacent OCT scans to a vortex vein ampulla demonstrate dilated, choroidal vessels that appeared to be connected to the vortex vein ampulla, supporting the hypothesis that ampullary engorgement may be affected by localized venous stasis [9,18].

Siddiqui and colleagues reported a case of a patient undergoing pars plana vitrectomy for vitreous opacities, in which an incidentally found vortex vein ampulla varicosity was noted [19]. The authors utilized intraoperative OCT, which demonstrated an elevation of the retinal pigment epithelium and neurosensory retina that reduced in size with scleral depression.

### 4.3. Literature on the Topic of Associated Systemic and Ocular Conditions

Although no ocular conditions were associated with vortex vein ampulla varicosities in our series, other reports have highlighted such associations. Zhang et al. [20] reported a patient referred for evaluation of a darkly pigmented choroidal mass. On exam, the patient had scleral injections and was diagnosed with scleritis. Funduscopic examination revealed a collapsible choroidal mass. The authors noted that this combination of scleral inflammation and a choroidal mass could be mistaken for melanoma, as melanoma may present with scleritis-like features. They further hypothesized that posterior scleritis-associated inflammation may impair choroidal venous outflow, resulting in transient engorgement of the vortex vein ampulla varix. This hypothesis was supported by the complete resolution of the varix following treatment of scleritis.

Similarly, Cabral et al. [21] reported a case of nodular posterior scleritis associated with a vortex vein ampulla varix. The lesion appeared hypopigmented on fundus exam, and OCT demonstrated a hyporeflective mass in the suprachoroidal space that remained engorged despite changes in gaze or application of ocular pressure. The authors hypothesized that nodular scleral changes may compress the adjacent choroid impeding venous outflow and inducing engorgement of the vortex vein ampulla varix. Unlike idiopathic cases, the presence of a fixed scleral nodule compressing venous outflow may have resulted in this persistent congestion.

Higham et al. [22] described two patients with Donnai–Barrow syndrome with vortex vein ampulla varices that were ectopically located in the macula. Additionally, these patients had extreme high myopia (>20 diopters). The authors discuss prior mouse models of this syndrome demonstrating that LRP2 mutations lead to abnormal RPE development secondary to impaired megalin expression. Because a healthy RPE is essential for the development of normal choroidal vasculature, this genetic defect likely results in congenital ectopic vortex veins and varices. While myopia has been linked to the development of acquired posterior vortex veins [3], the presence of these lesions in early infancy suggests a development abnormality inherent to the syndrome. Our study did not assess the relationship between refractive error/axial length and vortex vein ampulla varices, so we are unable to draw conclusions related to the presence of extreme high myopia in this study by Higham et al. [22] and vortex vein ampulla varices. In recent years, wide-angle indocyanine green angiography and optical coherence tomography–angiography (OCT-A) of the ocular fundus has demonstrated atypical choroidal vortex vein systems and ampullae located in the posterior fundus, including in juxtapapillary and macular sites, particularly in eyes with high axial myopia [2,3]. These posterior vortex vein ampullae are usually smaller than those that occur in the peripheral fundus and do not appear to be as prone to transient gaze induced congestion compared with their peripheral counterparts. Nevertheless, there are few reports of an occasional transient congestion of a posterior vortex vein ampulla [23,41].

Milani et al. [24] shared a highly myopic patient with a spontaneous suprachoroidal hemorrhage adjacent to a vortex vein ampulla varix. Although the hemorrhage resolved a few weeks later, the varix persisted. The authors suggested that engorged choroidal vessels, often seen in high myopia, may have predisposed the formation of a suprachoroidal hemorrhage.

Studies have associated the development of engorged vortex veins with pachychoroid spectrum diseases, including polypoidal choroidal vasculopathy and central serous chorioretinopathy. Chung et al. [42] hypothesized that in polypoidal choroidal vasculopathy, increased choroidal thickness and vascular hyperpermeability lead to venous congestion and subsequent engorgement of the associated vortex veins. While no published reports associate the pachychoroid spectrum disease specifically with varicosities of the vortex vein ampulla, and our series did not include measurements of choroidal thickness, this is a compelling area for future investigation. Investigations on this subject may provide further insight into the underlying pathophysiology of these lesions.

Weidmayer et al. [25] reported an intriguing case of a vortex vein ampulla varix that spontaneously appeared and then resolved. The authors hypothesized that the formation may have resulted from downstream thromboembolism within the vortex vein drainage system, though they noted that this was less likely given the patient’s concurrent anticoagulation therapy for atrial fibrillation. The authors also postulated that the resolution may be attributed to secondary choroidal venous anastomoses developed as a physiologic response to congestion. While these theoretical mechanisms are compelling, such interpretations require caution as they are speculative and are based on an isolated case of spontaneous lesion appearance and resolution.

## 5. Conclusions

Despite substantial research on vortex vein ampulla varicosities, particularly regarding the use of imaging modalities, several aspects of these lesions remain poorly understood. The relationship to refractive error, axial length, and systemic or ocular conditions has not been systematically studied. Additionally, the exact mechanism underlying gaze-induced venous congestion remains speculative. Future studies assessing the relationship between refractive error, axial length, and systemic or ocular conditions, such as pachychoroid spectrum disease, as well as investigations of choroidal blood-flow dynamics may refine our understanding of the factors influencing these lesions.

## Figures and Tables

**Table 1:** Categorical distribution of clinical variables evaluated in 53 patients with one or more varicosities of vortex vein ampulla.

Variable/Category	N (Number of Patients) (%)
Sex	
Male	34 (64.2)
Female	19 (35.8)
Age at initial diagnosis	
≤20 years	1 (1.9)
>20 years but ≤40 years	5 (9.4)
>40 years but ≤60 years	22 (41.5)
>60 years	25 (47.2)
Detection category of varicosity of vortex vein ampulla	
Detected by referring doctor and prompting referral	14 (26.4)
Detected during initial examination in ocular	
Oncology prompted by referral for unrelated	39 (73.6)
Lesion or abnormality	
Distribution of patients by lesion laterality	
Right eye only	29 (54.7)
Left eye only	21 (39.6)
Both eyes	3 (5.7)
Number of lesions in affected eye	
Single lesion in one affected eye	46 (86.8)
Two lesions in one affected eye	4 (7.5)
One lesion in each eye	2 (3.8)
Two lesions in each eye	1 (1.9)
Topographical location of central point of vascular lesion	
Superonasal quadrant	24 (38.7)
Inferonasal quadrant	24 (38.7)
Inferotemporal quadrant	10 (16.1)
Superotemporal quadrant	4 (6.5)
Largest basal diameter of congested vascular lesion	
≤2 mm	22 (35.5)
>2 mm but ≤3 mm	29 (46.8)
>3 mm but ≤4 mm	7 (11.3)
>4 mm	4 (6.4)
Smallest basal diameter of congested vascular lesion	
≤1.5 mm	28 (45.2)
>1.5 mm but ≤2.5 mm	26 (41.9)
>2.5 mm but ≤3.5 mm	7 (11.3)
>3.5 mm	1 (1.6)
Height of congested vascular lesion	
0.5 mm	3 (4.8)
1.0 mm	56 (90.3)
1.5 mm	3 (4.8)

**Table 2:** Cross-tabulation of direction of gaze that resulted in congestion of vascular lesion (columns) by location of vascular lesion (rows) for 61 varicosities of vortex vein ampulla ^a^.

	Direction of Ocular Gaze That Resulted in Vortex Vein Ampulla’s Congestion (N)								
Quadrant Location of Lesion	S	SN	N	IN	I	IT	T	ST	All
SN (superonasal)	5	18	0	0	0	0	0	0	1 ^b^
IN (inferonasal)	0	0	0	20	0	4	0	0	0
IT (inferotemporal)	0	0	0	0	1	9	0	0	0
ST (superotemporal)	0	1	0	0	0	0	0	2	0

Legend: N = number of patients, S = superior, SN = superonasal, N = nasal, IN = inferonasal, I = inferior, IT = inferotemporal, T = temporal, ST = supertemporal, ^a^ gaze direction that resulted in congestion of one superotemporal vortex vein ampulla was not recorded in patient’s chart, ^b^ one vortex vein ampulla remained persistently congested regardless of direction of gaze.

**Table 3:** Literature Review of Published Cases of Vortex Vein Ampulla Varicosities.

Study Author, Year [Ref]	Study Type	N (Patients)	N (Eyes)	Eye(s) Involved	Lesion Location	Multiplicity	Laterality	Dynamic Behavior	Advanced Imaging Modalities	Associated/Mimicked Conditions	Key Notes
Osher et al., 1981 [1]	Descriptive case series	5	5	Mixed (1 OD, 1 OU, 3 not specified)	Multiple (3 superonasal, 2 inferior, 1 not specified)	Single	One patient with bilateral	Gaze evoked intumescence; Valsalva evoked intumescence; collapse with globe pressure	B-scan, FA		
Buettner et al., 1990 [4]	Case report	1	1	OD	Superonasal	Single	Unilateral	Collapse with globe pressure; no change in intumescence with gaze change or positioning	B-scan	Choroidal melanoma mimic	Patient presenting with persistently engorged vortex vein ampulla varix.
Veronese et al., 2019 [5]	Descriptive imaging series	8	8	Mixed (7 OD, 1 OS)	Multiple	Single	Unilateral	Collapse with globe pressure	OCT, FA, ICGA, FAF, IR		
Ismail et al., 2011 [6]	Imaging case report	1	1	OD	Superonasal	Single	Unilateral	Collapse with globe pressure	OCT, ICGA	Subretinal hemorrhage mimic	
Vahdani et al., 2010 [7]	Case report	1	1	OS	Inferonasal	Multiple	Unilateral	Gaze evoked intumescence; collapse with globe pressure	Not described		
Suetov et al., 2022 [8]	Case series	2	Unable to assess—article not in English	Unable to assess—article not in English	Unable to assess—article not in English	Multiple	Bilateral	Unable to assess—article not in English	Unable to assess—article not in English		Article not in English so data abstraction relied on English-translated abstract.
Levin et al., 2025 [9]	Imaging case series	22	22	Mixed (12 OS, 10 OD)	Multiple locations reported	Single	Unilateral	Gaze evoked intumescence; valsalva evoked intumescence; collapse with globe pressure	OCT, B-scan, ICGA, IR		
Wolfensberger et al., 1997 [10]	Case report	1	1	OS	Superior	Single	Unilateral	Collapse with globe pressure	FA	Choroidal melanoma mimic	Article not in English, data abstraction relied on English-translated abstract. Unable to access complete article.
Kang et al., 2000 [11]	Imaging case report	1	1	OD	Superonasal	Single	Unilateral	Gaze evoked intumescence; collapse with globe pressure	FA, ICGA		
Levy et al., 2005 [12]	Case series	3	3	Mixed (2 OS, 1 OD)	Multiple (1 inferonasal, 2 superonasal)	Multiple	Unilateral	Gaze evoked intumescence; collapse with globe pressure	B-scan	Choroidal melanoma mimic	
Rimsaite et al., 2015 [13]	Case report	1	1	OS	Superotemporal	Single	Unilateral	Collapse with globe pressure	OCT, ICGA	Lesion simulated choroidal melanoma	Article not in English. Data transcribed using English-translated version.
Rubegni et al., 2025 [14]	Imaging case report	1	1	OS	Inferotemporal	Single	Unilateral	Collapse with globe pressure	OCT		
Shields et al., 1995 [15]	Descriptive imaging study	1	1	Not described	Not described	Single	Unilateral	Gaze evoked intumescence	ICGA		
de Carlo et al., 2021 [16]	Imaging case series	4	4	Mixed (2 OD, 2 OS)	Multiple (2 inferotemporal, 2 superonasal, 1 superotemporal)	One patient with multiple	Unilateral	Valsalva evoked intumescence; collapse with globe pressure	B-scan	Choroidal nevus, choroidal hemorrhage, and choroidal neoplasm mimic	
Spiess et al., 2022 [17]	Case report	1	1	OD	Superonasal	Single	Unilateral	Collapse with globe pressure	OCT		
Rayess et al., 2015 [18]	Imaging case report	1	1	OD	Superonasal	Single	Unilateral	Not described	OCT, B-scan, FA		Authors noted that OCT was the only imaging modality in this case that diagnosed the lesion.
Siddiqui et al., 2019 [19]	Imaging case report	1	1	OD	Inferonasal	Single	Unilateral	Collapse with globe pressure	Intraoperative OCT	Choroidal hemorrhage mimic during pars plana vitrectomy for vitreous opacities	
Zhang et al., 2018 [20]	Case report	1	1	OD	Superotemporal	Single	Unilateral	Collapse with globe pressure	B-scan	Anterior scleritis	
Cabral et al., 2022 [21]	Case report	1	1	OD	Inferior	Single	Unilateral	No change in intumescence with gaze change or digital pressure	OCT, B-scan, FA, ICGA	Possible association with nodular posterior scleritis	Patient presenting with persistently engorged vortex vein ampulla varix. After NSAID-treatment of posterior scleritis, lesion resolved.
Higham et al., 2022 [22]	Case series	2	4	OU	Macular	Multiple	Bilateral	Not described	OCT, ICGA	Association with Donnai–Barrow syndrome	
Ghadiali et al., 2017 [23]	Case report	1	1	OD	Superotemporal	Single	Unilateral	Collapse with globe pressure	OCT, ICGA	Choroidal melanoma mimic	
Milani et al., 2022 [24]	Case report	1	1	OD	Inferotemporal	Single	Unilateral	Not described	OCT, B-scan, FA ICGA	Suprachoroidal hemorrhage found adjacent to vortex vein ampulla varix; authors hypothesized that varix predisposed hemorrhage	
Weidmayer et al., 2021 [25]	Case report	1	1	OS	Superotemporal	Single	Unilateral	Collapse with globe pressure	OCT, B-scan, FAF		Lesion that spontaneously appeared and resolved, even when patient looked towards direction of lesion.
Snyers et al., 2002 [26]	Descriptive imaging series	Unable to assess—article not in English	Unable to assess—article not in English	Unable to assess—article not in English	Unable to assess—article not in English	Unable to assess—article not in English	Unable to assess due to article not being in English	Gaze evoked intumescence; Valsalva evoked intumescence	ICGA		Article not in English so data abstraction relied on English-translated abstract. Unable to access complete article.
Singh et al., 1993 [27]	Imaging case report	1	1	OS	Inferonasal	Single	Unilateral	Gaze evoked intumescence; Valsalva evoked intumescence	B-scan, ICGA	Lesion simulated choroidal melanoma	
Niu et al., 2025 [28]	Imaging case report	1	1	OD	Inferotemporal	Single	Unilateral	Gaze evoked intumescence; collapse with globe pressure	OCT, B-scan, ICGA		
Nitta et al., 2024 [29]	Imaging case report	1	1	OS	Inferonasal	Single	Unilateral	Gaze evoked intumescence; collapse with globe pressure	OCT, FA, ICGA		Patient with a vortex vein ampulla varix in OS and a macular vortex vein in OD.
Murtagh et al., 2021 [30]	Case report	1	1	OS	Superonasal	Single	Unilateral	Collapse with globe pressure	OCT		
Lopez et al., 1986 [31]	Case series	4	5	Mixed (1 OD, 2 OS, 1 OU)	Multiple (8 inferonasal, 3 superonasal)	Multiple	Bilateral and Unilateral	Gaze evoked intumescence; collapse with globe pressure	B-scan		
Khan et al., 2007 [32]	Prospective study	1	Not described	Not described	Not described	Not described	Not described	Not described	Not described	Choroidal melanoma mimic	Study assessing accuracy of referrals for suspected choroidal melanoma.
Kang et al., 2017 [33]	Imaging case report	1	1	OD	Superonasal	Single	Unilateral	Gaze evoked intumescence; collapse with globe pressure	OCT, FA, ICGA		Description of vortex vein ampulla dynamics using real-time angiography.
Hunter et al., 1983 [34]	Case series	2	2	Mixed (1 OD, 1 OS)	Multiple (1 superonasal, 1 superior)	Single	Unilateral	Gaze evoked intumescence; collapse with globe pressure			
Gunduz et al., 1998 [35]	Case series	4	4	Not described	Multiple	One patient with multiple varix	Unilateral	Gaze evoked intumescence	B-scan, FA, ICGA	Choroidal melanoma mimic	
Garcia-Bardera et al., 2024 [36]	Imaging case report	1	1	OD	Superonasal	Single	Unilateral	Gaze evoked intumescence; valsalva evoked intumescence; collapse with globe pressure	OCT		Article not in English so data abstraction relied on english-translated abstract. Unable to access complete article.
da Cruz et al., 1994 [37]	Case report	1	1	OD	Multiple (1 superior, 1 superonasal)	Multiple	Unilateral	Gaze evoked intumescence; position dependent; collapse with globe pressure.		Choroidal metastases mimic	
Al-Dahmash et al., 2021 [38]	Case report	1	Unable to assess—article not in English	OD	Unable to assess—article not in English	Multiple	Unable to assess—article not in English	Unable to assess—article not in English	Unable to assess—article not in English	Choroidal metastases mimic	Article not in English so data abstraction relied on english-translated abstract. Unable to access complete article.

Abbreviations: FA, fluorescein angiography; FAF, fundus autofluorescence; ICGA, indocyanine green angiography; IR, infrared; OCT, optical coherence tomography.

## Data Availability

The datasets presented in this article are not readily available because the data are part of an ongoing study under IRB supervision. Requests to access the datasets should be directed to James J. Augsburger, MD [augsbujj@ucmail.uc.edu].

## Abbreviations

The following abbreviations are used in this manuscript:

B-scan	Standard B-scan ultrasound
ICGA	Indocyanine green angiography
FA	Fluorescein angiography
OCT	Optical coherence tomography
OCT-A	Optical coherence tomography–angiography

## References

[1] Osher R.H., Abrams G.W., Yarian D., Armao D. (1981). Varix of the vortex ampulla. Am. J. Ophthalmol..

[2] Verma A., Maram J., Alagorie A.R., Gupta Nittala M., van Hemert J., Keane D., Carnevale J., Bell D., Singer M., Sadda S.R. (2020). Distribution and Location of Vortex Vein Ampullae in Healthy Human Eyes as Assessed by Ultra-Widefield Indocyanine Green Angiography. Ophthalmol. Retin..

[3] He G., Zhang X., Zhuang X., Zeng Y., Gan Y., Su Y., Li M., Ji Y., Mi L., Chen X. (2024). A Novel Exploration of the Choroidal Vortex Vein System: Incidence and Characteristics of Posterior Vortex Veins in Healthy Eyes. Investig. Ophthalmol. Vis. Sci..

[4] Buettner H. (1990). Varix of the vortex ampulla simulating a choroidal melanoma. Am. J. Ophthalmol..

[5] Veronese C., Staurenghi G., Pellegrini M., Maiolo C., Primavera L., Morara M., Armstrong G.W., Ciardella A.P. (2019). Multimodal Imaging in Vortex Vein Varices. Retin. Cases Brief Rep..

[6] Ismail R.A., Sallam A., Zambarakji H.J. (2011). Optical coherence tomographical findings in a case of varix of the vortex vein ampulla. Br. J. Ophthalmol..

[7] Vahdani K., Kapoor B., Raman V.S. (2010). Multiple vortex vein ampulla varicosities. BMJ Case Rep..

[8] Suetov A.A., Boiko E.V., Izmaylov A.S., Molodkina N.A. (2022). [Vortex vein varix (clinical observations)]. Vestn. Oftalmol..

[9] Fogel Levin M., Hostovsky A., Fossataro C., Caputo C.G., Feo A., Romano M.R., Fossataro F., Fried S., Birger Y., Moroz I. (2025). Vortex Vein Varix: Multimodal Imaging and Clinical Correlates. Ophthalmol. Retin..

[10] Wolfensberger T.J. (1997). [Varix of the vortex ampulla: An unusual differential choroid tumor diagnosis]. Klin. Monbl. Augenheilkd..

[11] Kang H.K., Beaumont P.E., Chang A.A. (2000). Indocyanine green angiographic features of varix of the vortex vein ampulla. Clin. Exp. Ophthalmol..

[12] Levy J., Yagev R., Shelef I., Lifshitz T. (2005). Varix of the vortex vein ampulla: A small case series. Eur. J. Ophthalmol..

[13] Rimsaite A., Andersen C.U. (2015). [A varix of the vortex vein ampulla is a rare differential diagnosis of malignant melanoma of the choroid]. Ugeskr. Laeger.

[14] Rubegni G., Bacci T., Tosi G. (2025). Vortex Vein Varix changes with digital pressure: Ultra-Wide-Field imaging and Peripheral OCT report. Am. J. Ophthalmol..

[15] Shields C.L., Shields J.A., De Potter P. (1995). Patterns of indocyanine green videoangiography of choroidal tumours. Br. J. Ophthalmol..

[16] de Carlo T.E., Mieler W. (2021). Dynamic Echography of Varix of the Vortex Vein Ampulla. Retin. Cases Brief Rep..

[17] Spiess K., Elgohary M.A. (2022). Diagnosis of Vortex Varix Using Optical Coherence Tomography and Scleral Indentation. Retin. Cases Brief Rep..

[18] Rayess H., Ehlers J.P. (2015). Utilization of peripheral optical coherence tomography to optimize diagnosis of a vortex vein varix. Retina.

[19] Siddiqui M.Z., Sanders R., Sallam A.B. (2019). Utilization of Intraoperative OCT for the Diagnosis of a Case of Varix of the Vortex Vein. Ophthalmol. Retin..

[20] Zhang X., Olson D.J., DiBernardo C., Davis R.M., Gordon K.G.B. (2018). Scleritis-associated vortex vein varix masquerading as choroidal melanoma. Can. J. Ophthalmol..

[21] Cabral D., Nogueira V. (2022). Varix of a Vortex Vein Ampulla Induced by Nodular Scleritis. Retin. Cases Brief Rep..

[22] Higham A., Hildebrand G.D., Graham-Evans K.A.J., Gilbert R.D., Horton R., Hunt D., Shears D., Patel C.K. (2022). Ectopic vortex veins and varices in Donnai Barrow syndrome. Ophthalmic Genet..

[23] Ghadiali Q., Tan A., Freund K.B. (2017). Unusual Posterior Varix of a Vortex Vein Ampulla. Retin. Cases Brief Rep..

[24] Milani P., Mazzola M., Bergamini F. (2022). Suprachoroidal haemhorrage and vortex vein varix: A potential association. Eur. J. Ophthalmol..

[25] Weidmayer S.L., Demirci H. (2021). The spontaneous resolution of a vortex vein varix: Case report. BMC Ophthalmol..

[26] Snyers B., De Potter P. (2002). [Advantages of digital indocyanine green angiography for diagnosing choroidal tumors]. J. Fr. Ophtalmol..

[27] Singh A.D., De Potter P., Shields C.L., Shields J.A. (1993). Indocyanine green angiography and ultrasonography of a varix of vortex vein. Arch. Ophthalmol..

[28] Niu T.T., Xiao Y. (2025). Varix of the vortex vein ampulla: A case report and imaging correlation. Front. Med..

[29] Nitta K., Akiyama H. (2024). Different Vortex Vein Anomalies Observed in a Single Case: Macular Vortex Vein in One Eye and Varix of Vortex Vein Ampulla in the Other Eye. Cureus.

[30] Murtagh P., O’Dwyer G., Horgan N. (2021). Vortex Vein Ampulla. Ophthalmology.

[31] Lopez P. (1986). Varix of the vortex vein ampulla. J. Am. Optom. Assoc..

[32] Khan J., Damato B.E. (2007). Accuracy of choroidal melanoma diagnosis by general ophthalmologists: A prospective study. Eye.

[33] Kang T.D., Douglass A.M., Ferenczy S.R., Say E.A., Shields C.L. (2017). In Vivo Hemodynamic Changes of Vortex Vein Varix on Real-Time Video Angiography. Retina.

[34] Hunter J.E. (1983). Vortex vein varix. Am. J. Optom. Physiol. Opt..

[35] Gunduz K., Shields C.L., Shields J.A. (1998). Varix of the vortex vein ampulla simulating choroidal melanoma: Report of four cases. Retina.

[36] Garcia-Bardera J., Montolio-Marzo E., Etxabe-Avila H., Lorenzo-Castro J., Garcia-Caride S. (2024). Valsalva-induced changes in vortex vein varices: A comprehensive imaging exploration. J. Fr. Ophtalmol..

[37] da Cruz L., James B., Gray R., Elston J. (1994). Multiple vortex vein varices masquerading as choroidal secondaries. Br. J. Ophthalmol..

[38] Al-Dahmash S.A., AlBloushi A.F., Alsarhani W.K. (2021). Multiple giant vortex vein varices masquerading as choroidal metastases. J. Fr. Ophtalmol..

[39] Rutnin U. (1967). Fundus appearance in normal eyes: I. The choroid. Am. J. Ophthalmol..

[40] Hu Y., Wang S., Dong Y., Zhou X., Yu W., Xu C. (2011). Imaging Features of of Varix of the Vortex Vein Ampulla: A Small Case Series. J. Clin. Exp. Ophthalmol..

[41] Gass J.D. (1983). Uveal effusion syndrome. A new hypothesis concerning pathogenesis and technique of surgical treatment. Retina.

[42] Chung S.E., Kang S.W., Kim J.H., Kim Y.T., Park D.Y. (2013). Engorgement of vortex vein and polypoidal choroidal vasculopathy. Retina.

